# An alternative assessment pathway: Lessons from Australia and New Zealand

**DOI:** 10.1192/j.eurpsy.2023.715

**Published:** 2023-07-19

**Authors:** V. Lakra

**Affiliations:** Royal Australian and New Zealand College of Psychiatrists, Melbourne, Australia

## Abstract

**Introduction:**

The COVID-19 pandemic disrupted specialist training across Australia and New Zealand particularly in the area of assessments. Large-scale face-to-face exams were especially vulnerable to disruptions including lockdowns, travel and density restriction and required the Royal Australian and New Zealand College of Psychiatrists (RANZCP) to introduce alternatives.

Following several small-scale Objective Structured Clinical Exams (OSCEs) in late 2020 and 2021 (both AV and multi-site), the RANZCP introduced an Alternative Assessment Pathway (AAP) after an exam cancellation in November 2021.

**Objectives:**

To discuss the introduction of an Alternative Assessment Pathway at the RANZCP and share progress regarding the broader changes to assessment models under consideration.

**Methods:**

The AAP was co-designed with trainees and Specialist International Medical Graduates and introduced in December 2021 as an interim measure to assess clinical competence in the absence of an OSCE. It comprises a portfolio review of completed end-of-rotation forms and, if required, a case-based discussion held via Zoom.

**Results:**

The AAP has been held over two rounds (December 2021 and March 2022) with 97% and 90% pass rates respectively (data correct as at 6 October 2022). An evaluation into the pathway is currently underway.

In July 2022, the RANZCP introduced the Clinical Competency Assessment as a continuation of the AAP (with some modifications) for the remainder of 2022 and 2023.

**Conclusions:**

While the pandemic has become a catalyst for change, the RANZCP has been considering broader changes to the theory and practice of its assessments for some time. The presentation will provide an overview of its short-term clinical assessment model and share progress regarding change to the long-term assessment strategy.

**Disclosure of Interest:**

None Declared

